# BUILDing pathways to health-related research careers in biomedical and behavioral sciences: a longitudinal evaluation of postbaccalaureate outcomes using a matched comparison group

**DOI:** 10.3389/feduc.2025.1474224

**Published:** 2025-01-27

**Authors:** Erin H. Arruda, Kim-Phuong L. Vu, Chi-Ah Chun, Gino Galvez, Panadda Marayong, Jesse G. Dillon

**Affiliations:** 1 Department of Educational Leadership, California State University, Long Beach, Long Beach, CA, United States,; 2 Department of Psychology, California State University, Long Beach, Long Beach, CA, United States,; 3 Department of Mechanical and Aerospace Engineering, California State University, Long Beach, Long Beach, CA, United States,; 4 Department of Biological Sciences, California State University, Long Beach, Long Beach, CA, United States

**Keywords:** biomedical, workforce, stem, education, undergraduate research, doctorate, research skills, careers in science

## Abstract

The BUilding Infrastructure Leading to Diversity (BUILD) undergraduate research training program is funded by the National Institutes of Health (NIH) to strengthen the pipeline for underrepresented students through graduate school and into health-related research careers in the biomedical and behavioral sciences. This study evaluates the impact of BUILD participation at a Minority-Serving Institution in Southern California on graduate school outcomes up to 6 years post-graduation including doctoral program enrollment and degree attainment using a quasi-experimental design. BUILD students were compared to a propensity score matched non-BUILD group using logistic regression. Results showed BUILD students enrolled in Ph.D. programs and attained Ph.Ds. at a higher rate compared to matched peers. Findings indicate BUILD met a pivotal program objective to increase doctoral degree attainment imperative for health-related research careers in biomedical and behavioral sciences. Furthermore, results support the added value of undergraduate research programs for students from underrepresented backgrounds.

## Introduction

Scientists and researchers who identify as underrepresented races/ethnicities or as women often face barriers at critical transition points through the postbaccalaureate pipeline (Bennett et al., 2020a) and faculty pathway (Ransdell et al., 2021). To increase diversity of perspectives and backgrounds in the biomedical and behavioral health sciences (BBHS) research workforce and professoriate, increased diversity in doctoral degree attainment in related fields must be achieved. This means that diversity in enrollment in doctoral degree programs in these fields is paramount. Efforts to increase representation of traditionally underrepresented groups in postbaccalaureate enrollments and degree attainments in STEM fields include evidence-based interventions at the pre-doctoral level such as undergraduate research training programs (Bayliss et al., 2018; Hall et al., 2016; Vu et al., 2023). Training programs that support strengthening the pipeline to BBHS research careers for underrepresented groups have strong commitments to diversity, equity, inclusion (DEI) and social justice. In recent years, there has been an increase of anti-affirmative action movements which could have negative impacts on efforts to accelerate the enhancement of diversity of educational and employment spaces where groups have been explicitly and implicitly excluded due to racism and racist policies. Therefore, it is now more crucial than ever to demonstrate the effectiveness of undergraduate research training programs that are open to all students and include intentional components designed to attract and support students who are traditionally underrepresented in higher education and sciences.

Research on interventions to broaden the research workforce in BBHS demonstrates positive short-term outcomes (e.g., Kingsford et al., 2022). However, methodological concerns (Linn et al., 2015) and limited data on longer-term outcomes (e.g., post-baccalaureate education and career status) warrant caution in extrapolating these findings to longer-term impacts. Specifically, studies on long-term effectiveness of programs have typically relied on self-report data, used inadequate comparison groups, and/or focused on short-term (e.g., undergraduate GPA, psychosocial constructs) or broad, longer-term goals (any graduate-level acceptance or degree attainment) rather than doctoral degrees specific to the BBHS (see Whittinghill et al., 2019 for exception, although science was broadly defined as STEM and degree attainment was not included). Unfortunately, these limitations are significant because they assume that underrepresented students who receive strong training and mentorship during their undergraduate years will naturally continue to complete a doctoral program and earn a Ph.D. degree. Yet, a recent NSF report (2024) revealed that in 2023, underrepresented minority (URM) students earned 28% of bachelor’s degrees in science and engineering, but only 19.4% of doctoral degrees (while 67.4% of bachelor’s degrees and 77.1% of doctorates were earned by non-URM students). Moreover, Bennett et al. (2020a) reported that only 10.1% of faculty at 4-year universities are from URM backgrounds. These findings indicate that disproportionately smaller numbers of URM undergraduate students in STEM move on to doctoral programs and even fewer attain faculty positions. Additionally, Bennett et al. (2020b) found little evidence of change in race/ethnicity and gender make-up of faculty of basic science faculty across U.S. medical schools.

To examine longer-term outcomes of research training interventions and evaluate the effectiveness of an intensive undergraduate research training program, the current study employed a quasi-experimental design to examine BBHS-specific postbaccalaureate milestones including enrollment and degree attainment up to 6 years post-undergraduate graduation. The research questions for this study were addressed using a large, multi-year sample from a BUILD undergraduate research training program at California State University, Long Beach (CSULB), a diverse, urban, R2 university. Funded by the National Institutes of Health (NIH), the CSULB BUILD Program aims to strengthen the pipeline for underrepresented students through graduate school and into health-related research careers in the biomedical and behavioral sciences. This study included a comparison group, constructed with propensity score matching, to compare undergraduate students who participated in BUILD to undergraduate students who did not participate in BUILD but were similar on a variety of demographics and other characteristics.

**FIGURE 1:**
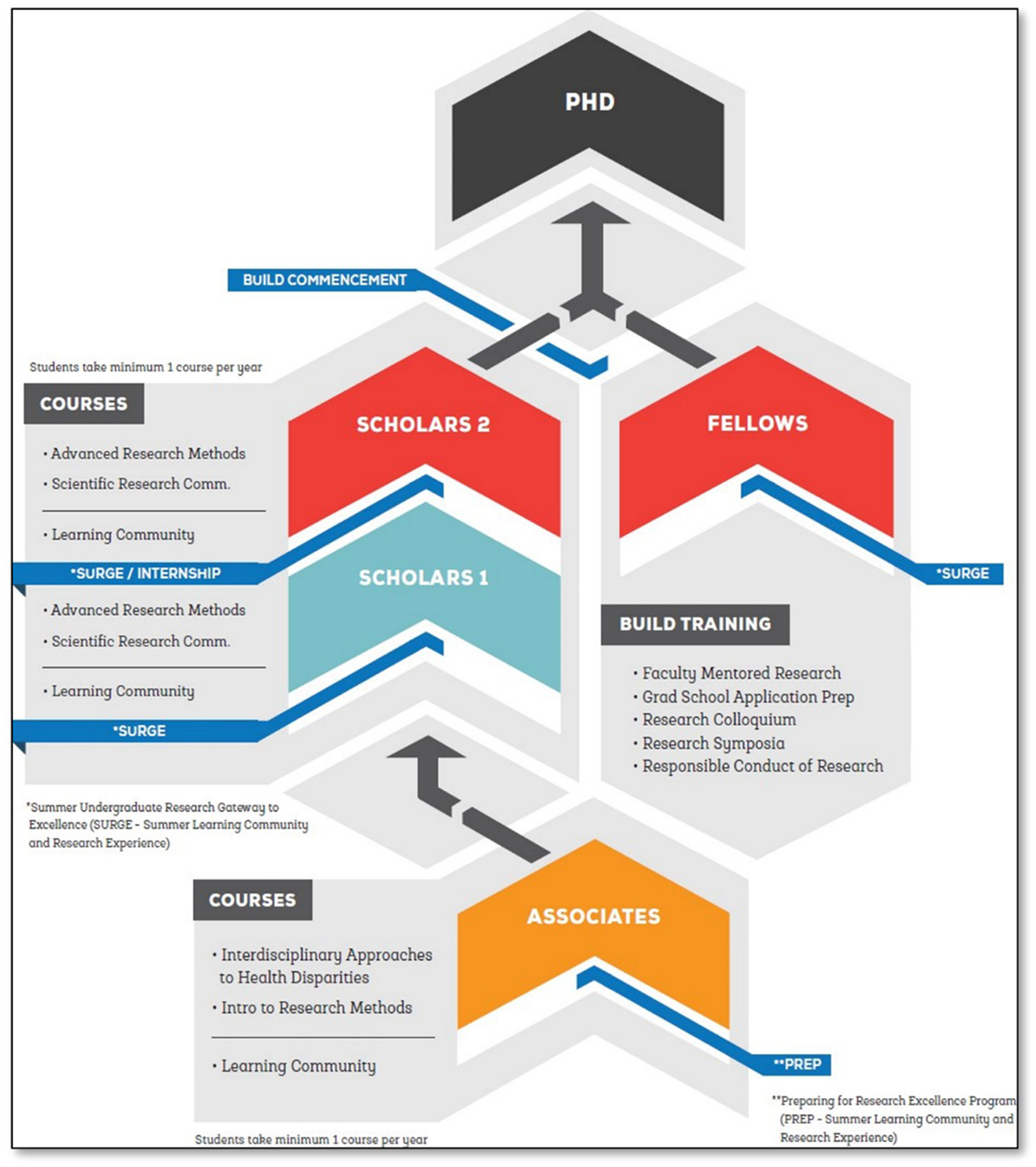
Structure of the BUILD program cohorts (Associates, Scholars, and Fellows) and timeline.

The CSULB BUILD Program is unique as it is large in scale and aimed specifically at undergraduate research training in health-related disciplines, broadly defined across four different colleges of Engineering, Health and Human Services, Liberal Arts, and Natural Sciences and Mathematics. It is a cohort-based model, with targeted activities based on the year that students entered the program (see Figure 1). For example, the Associates program was designed as an early intervention program for sophomore-level students (see Kingsford et al., 2022 for details) with the goal of developing their interest in pursuing research at the upper division level. About 62% of Associates were accepted into one of three NIH-funded upper-division research training programs at CSULB (BUILD Scholars, MARC U*STAR, and RISE), with 54% continuing with the BUILD Scholars program. The Scholars program, which is described in more detail below, targets juniors with four semesters left before graduation. The 1-year Fellows program targets seniors in their last undergraduate year. The application and interview processes are the same for all three BUILD programs (see Cho et al., submitted). Moreover, all three programs include a summer research training component, support for faculty-mentored research during the year, and professional development in a learning community cohort.

The Scholars Program is an intensive 2-year program. It begins with an 8-week summer program, where students meet in a learning community twice a week for a total of 6 h to engage in general research skills training (e.g., how to read a research article, developing research questions and hypothesis, and ethics training) and professional development activities (e.g., pathway to graduate school, “elevator speeches,” and poster presentations). BUILD faculty training directors lead the learning community with the support of graduate mentors. Scholars spend the rest of the week (~30–34 h) working on research projects mentored by faculty members in their discipline. The summer program culminates with a research symposium where the trainees highlight their summer accomplishments via poster presentations to their faculty mentors, lab mates, families, and friends. During the academic year of Year 1, Scholars continue to work on their research projects with their faculty mentors and participate in a learning community for 1 h per week. Trainees are encouraged to attend conferences geared toward undergraduates from underrepresented backgrounds (e.g., SACNAS or ABRCMS) in the fall. Travel support is provided by BUILD to reduce financial barriers and give students the opportunity to develop their networking skills and engage with other students, researchers, and graduate program recruiters. During Year 1, Scholars are required to apply for summer research experience programs at an R1 university. Students who are not accepted or who decide not to go to an external summer research experience program continue to work with their BUILD faculty mentors over the summer and engage in the summer learning community alongside 1st-year students (with different breakout groups). During their second academic year (Year 2), Scholars continue to work with their faculty mentors on disciplinary research, receive travel funds to present their research at a professional conference in their discipline, and are mentored as they apply to graduate school. Year 2 Scholars celebrate their conclusion of the program with a commencement ceremony. The Fellows Program is intended for seniors with prior research experience but may not have known about the BUILD Program until their senior year and did not participate in the Scholar program in the prior year. Fellows participate in the 8-week summer research training component jointly with the Year 1 Scholars during the summer and engage in similar training activities as the Year 2 Scholars during the academic year.

Throughout their entire experience in the program, BUILD trainees receive programmatic mentoring (mentoring by BUILD faculty training directors and near-peer graduate mentors, see Abeywardana et al., 2020) to increase their research skills, sense of belonging, and motivation to persist in research careers. Scholars also receive significant financial support (i.e., monthly stipend, research supply fund, and travel funds) that allows them to focus on their research and educational requirements rather than seek out external employment (see Vu et al., 2023 for more detailed information about the CSULB BUILD Scholars Program). The Associates and Fellows receive hourly pay, rather than a stipend, for their research training along with research supplies and travel funds because those programs are funded under a different NIH award mechanism.

This study aims to determine whether the CSULB BUILD Student Training Programs are effective in creating a direct pathway for students from the bachelor’s to Ph.D. to maximize the potential for increasing diversity in the BBHS research workforce. The following three research questions are considered for BUILD students (defined as Associates, Scholars and Fellows who started the program during the 2015–2018 academic years after completing the summer component).

Question 1: Are BUILD students enrolled in doctoral programs at a higher rate than their matched non-BUILD peers after graduating from CSULB? Are BUILD students enrolled in fields associated with the BBHS research career?

Question 2: Do BUILD students earn doctoral degrees at a higher rate than their matched non-BUILD peers? Are BUILD students’ doctoral degree types (Ph.D. vs. professional doctoral degrees) different compared to those of their matched peers? Are BUILD students earning degrees in fields associated with the BBHS research career?

Question 3: Are there differences in BUILD doctoral degree outcomes by identities (i.e., gender, and race/ethnicity) and family educational status (i.e., first-generation) compared to those of the matched non-BUILD peers?

## Materials and methods

### Participants

The pre-matched sample pool included 281 BUILD students (comprised of Associates, Scholars, and Fellows) and 6,310 non-BUILD students at CSULB. See Table 1 for demographic characteristics of the two student groups before matching. BUILD students were from four cohorts (2015–2018) and entered the program as sophomores (Associates, 47.2%), juniors (Scholars, 50.2%), or seniors (Fellows, 2.6%).

### Overview of matching process

Propensity score matching (PSM, Bai and Clark, 2019; Rosenbaum and Rubin, 1985) method was used to create a matched comparison group of non-BUILD to BUILD students. Propensity scores are predicted probabilities for selection into BUILD using predictors as matching variables in a statistical model. A PSM approach is efficient when it is cumbersome to match participants on many variables at the same time. Conceptually, matches of non-BUILD students are selected that have the same or very similar propensity scores as BUILD students. To accomplish the matching process, we used predictor variables that related to the selection into the BUILD training program. After the matching procedure and omitting data of non-matched students, statistical analyses were performed to address the research questions comparing the BUILD group to the matched non-BUILD group on outcomes such as doctoral program enrollment and degree attainment as described below.

### Covariate selection

A pool of covariates was constructed from programmatic selection criteria of BUILD. In addition to all demographic variables listed in Table 2, the GPA before BUILD participation and participation in independent research were also included as potential covariates. All data were retrieved from the Office of Institutional Research and Analytics at CSULB. Next, statistical relationships were assessed between each covariate and BUILD participation. Associations were also assessed between each covariate and the last recorded GPA and graduation status. Final decisions about which covariates were included in the PSM model were based on effect sizes of *t*-tests, chi-square, or correlation tests (as appropriate for the levels of measurement). Cohen’s *d* > 0.10 or *r* > 0.10 indicated that a covariate was not balanced between BUILD and non-BUILD groups and therefore should be included in the PSM model. For statistical tests between the variables GPA and graduation status and potential covariate, a *d* > 0.10 or *r* > 0.10 indicated a covariate was sufficiently related to the outcome to also be considered for inclusion in the PSM model.

Pre-matching results are displayed in Table 2, Column 2. Several variables differed by BUILD participation status, including age, year of entry (CSULB matriculation), full-time status, transfer status, college, GPA at entry to CSULB and independent research experience, and were therefore included in the PSM model. Although other variables appeared balanced between BUILD and non-BUILD groups, results showed that these variables were related to GPA and/or graduation status (see Columns 3 and 4 in Table 2) and consequently could influence the way BUILD participation impacts graduate school outcomes. Therefore, these variables were also included in the PSM (see for example, gender). For variables that were initially balanced between BUILD and non-BUILD groups, checks were done post-matching to confirm that balance was maintained.

### Estimation of propensity scores

Nearest neighbor matching (1:1) was selected because it is an efficient and commonly used method based on the closest absolute distance between propensity scores. Caliper matching was applied as it is suited for a large comparison group. Following the formula by Rosenbaum and Rubin (1985), a caliper width of 0.035 was calculated based on *c* = 0.25 (SD[p(x)]), where SD = standard deviation of the propensity scores (here, 0.14) to remove at least 90% of bias (Bai, 2011; Cochran and Rubin, 1973). Due to good common support (that is, non-BUILD group propensity scores overlap with most of the BUILD group) and given that the non-BUILD group was more than three times the size of the BUILD group, replacement was not needed. First, data were prepared for logistic regression. Variables that were categorical with more than two categories were dummy coded. When categories had a frequency of zero for BUILD and very small frequency for non-BUILD (<5%), non-BUILD participants from these categories were excluded to avoid estimation problems. This included Native American and Pacific Islander categories for race/ethnicity and the College of Business Administration and College of Education categories. White was the referent group for race/ethnicity and College of Liberal Arts was the referent group for college. Collinearity was also checked between predictors because perfect collinearity will lead to non-convergence and high collinearity could result in “bouncing betas” including large standard errors, and implausible estimates. Tolerance and VIF values indicated no issues with perfect or high multicollinearity. A logistic regression with BUILD status as the outcome was estimated to examine the overall model fit, (χ^2^ [21, *N* = 4,820] = 988.26, *p* < 0.001, Nagelkerke *R*^2^ = 0.52). The model converged in nine iterations. Estimates are presented in Table 3.

### Evaluation of matching

Covariates could remain unbalanced after matching, and matching could potentially increase imbalance. Therefore, each covariate was examined for balance, even those that appeared balanced prior to matching. Matching for referent groups for categorical variables was also checked. Balance was assessed by examining the magnitude of difference for each covariate between BUILD and non-BUILD groups. Standardized mean differences were calculated (SMD, based on Cohen’s *d*). Standardized bias was calculated by multiplying the SMD by 100. A standardized bias <10% reflects adequate balance (coinciding with Cohen’s *d* < 0.10; Bai and Clark, 2019). These estimates appear in Table 2, Column 5. All SMDs are lower than the recommended threshold of 0.10, indicating that standardized bias is <10% for each covariate and all relationships are non-significant. Given the level of balance achieved, the PSM model was adequate and was used to examine possible differences in outcomes between BUILD and their matched non-BUILD students. The same matched groups were used to address each research question in this study. Simulation evidence supports the use of a single, generic-outcome propensity score model when multiple, related outcomes are examined (Wyss et al., 2013).

### Measures

Outcome data were retrieved from the National Student Clearinghouse (NSC) in late September 2024 to track student graduate outcomes. Variables included graduate program enrollment, enrollment major and/or the Classification of Instructional Programs (CIP; a major code), degree attainment, degree major and/or CIP codes, and type of degree attained (e.g., Ph.D., Professional doctoral degrees such as Doctor of Physical Therapy, Optometry, or Pharmacy). All variables were binary coded (e.g., 1 = enrolled, 0 = not enrolled; 1 = Ph.D. earned, 0 = No Ph.D. earned). BBHS-related majors were coded 1 (e.g., chemistry, neuropsychology), while non-BBHS related majors were coded 0 (e.g., geography, English). Classification decisions were reviewed by two independent reviewers. Regarding enrolled Ph.D. program disciplines, CIP codes and/or majors were reported for 77% of students enrolled. Major information was missing at a slightly higher rate for the non-BUILD students than for the BUILD students (29% vs. 21% missing, respectively). When all missing majors were imputed based on other information provided (e.g., type of doctorate or master’s degree earned, and their major in another program), no substantive differences in results were found. Therefore, we used the imputed CIP/major codes for completeness. Majors were further categorized as either biomedical (i.e., natural sciences, specific engineering type majors such as biomedical engineering, chemistry, coded 1) or behavioral disciplines (i.e., liberal arts, specific health and human services type majors such as psychology or physical therapy, coded 0). BUILD status was binary coded (i.e., 1 = BUILD student, 0 = non-BUILD student).

### Data analysis

IBM SPSS (v.29; IBM Corp, 2023) was used for all analyses. Binary logistic regression models were estimated due to binary outcome variables. Some student records were not obtained. Per NSC, data are missing when students have their records blocked or partially blocked, when student records were not found, or when an institution does not report data to NSC. With <7% of data missing, this reduced the total sample size to 495 (*N*_BUILD_ = 268, *N*_Non–BUILD_ = 227). We reexamined the evaluation of matched groups based on this reduced sample size due to missing data. The analysis reflected that missingness did not have an impact on the matching of the groups, and thus, groups were still well matched (see Table 2). Odds ratios (OR), a natural effect size, were reported and interpreted when the predictor was statistically significant. The Nagelkerke R-squared (*R*^2^*_N_*) was also reported as an effect size. The *R*^2^*_N_* was selected as a pseudo-*R*^2^ because it ranges from 0 to 1, with higher values indicating larger effect sizes. If the expected count for a category was very small (e.g., <5), a Fisher’s Exact test was analyzed instead. All inferential tests were two-tailed tests (*α* = 0.05).

## Results

### Research question 1

Are BUILD students enrolled in doctoral programs at a higher rate than their matched non-BUILD peers after graduating from CSULB? Are BUILD students enrolled in fields associated with the BBHS research career?

Counts of doctoral enrollment by BUILD program participation status are displayed in Table 4. Overall, there were 128 total enrollments (25.9%) in doctoral programs (86 BUILD student enrollments and 42 non-BUILD student enrollments). There was a statistically significant difference in the number of doctoral enrollments (regardless of type) by BUILD status, *X*^2^(1) = 12.06, *p* < 0.001, $R_{N}^{2}=0.04$. The odds of enrolling in any doctoral degree program for BUILD students were 2.08 times greater than the odds for non-BUILD matched students, *b* = 0.73, *SE* = 0.22, *OR* = 2.08, *p* < 0.001. Moreover, 88 students (17.8% of all students) were enrolled in a Ph.D. granting graduate program. A little more than a quarter (26.5%, *N* = 71) of BUILD students and 7.5% (*N* = 17) of non-BUILD students were enrolled. The difference in enrollment in Ph.D. program between the two groups was statistically significant, *X*^2^(1) = 32.63, *p* < 0.001, $R_{N}^{2}=0.11$. For BUILD students the odds of enrollment in a Ph.D. program were 4.45 times the odds for non-BUILD students, *b* = 1.49, *SE* = 0.29, *OR* = 4.45, *p* < 0.001. Regarding the disciplines, a slightly higher proportion of BUILD students enrolled in BBHS-related disciplines than non-BUILD students (93.0%, *N* = 66; 82.4%, *N* = 14, respectively). However, a Fisher’s Exact test indicated a non-significant association between BUILD status and the BBHS-related discipline of Ph.D. program enrolled, *p* = 0.180. Within the BBHS-related disciplines, 71.2% (*N* = 47) of the BUILD Ph.D. enrollments were in biomedical disciplines while 28.8% (*N* = 19) were in behavioral disciplines. For the 14 non-BUILD students enrolled in Ph.D. programs within the BBHS-disciplines, 85.7% (*N* = 12) were in biomedical disciplines while 14.3% (*N* = 2) were in behavioral. A Fisher’s Exact test indicated that Ph.D. enrollment discipline types for BBHS enrollments did not differ between BUILD and non-BUILD students, *p* = 0.334.

A small percentage of students were enrolled in professional degree granting programs (7.9%, *N* = 40) with a higher percentage of non-BUILD students (11.1%, *N* = 25) attending professional programs than BUILD students (5.6%, *N* = 15). There was a significant difference in professional degree program enrollment by BUILD status, *X*^2^(1) = 4.86, *p* = 0.028, $R_{N}^{2}=0.02$. The odds of BUILD students enrolling in a professional degree program were about half the odds of non-BUILD students (*b* = −0.74, *SE* = 0.34, *p* = 0.030, *OR* = 0.48). Of the 15 BUILD professional degree program enrollments, 93.3% (*N* = 14) were in BBHS-related majors. Of the 25 enrollments for non-BUILD students, 88.0% (*N* = 22) were in BBHS-related majors. A Fisher’s Exact test indicated a non-significant association between BUILD status and enrollment BBHS-related major for the professional degree program enrollments, *p* = 0.999.

### Research question 2

Do BUILD students earn doctoral degrees at a higher rate than their matched non-BUILD peers? Are BUILD students’ doctoral degree types (Ph.D. vs. professional doctoral degrees) different compared to those of their matched peers? Are BUILD students earning degrees in fields associated with the BBHS research career?

Counts of degrees attained by BUILD program participation status are displayed in Table 5. Overall, 42 doctorate degrees were earned by late September 2024 (20 Ph.Ds. and 22 professional degrees). BUILD students earned a total of 30 doctorate degrees (18 Ph.Ds. and 12 professional degrees) while non-BUILD students earned 12 doctorate degrees (2 Ph.Ds. and 10 professional degrees). Logistic regression results indicated that there was a statistically significant difference in number of doctorate degrees (regardless of type) earned by BUILD participation status, *X*^2^(1) = 5.74, *p* = 0.017, $R_{N}^{2}=0.03$. The odds of earning any doctoral degree for BUILD students were 2.26 times greater than the odds for matched non-BUILD students, *b* = 0.82, *SE* = 0.35, *OR* = 2.26, *p* = 0.021. Additionally, there was a statistically significant difference in Ph.D. attainment by BUILD status, *X*^2^(1) = 12.64, *p* < 0.001, *R*^*2*^_*N*_ = 0.09. The odds of earning a Ph.D. were 8.10 times for BUILD students compared to matched non-BUILD students, *b* = 2.09, *SE* = 0.75, *OR* = 8.10, *p* = 0.005. Lastly, there was not a statistically significant difference in professional degree attainment by BUILD status, *X*^2^(1) = 0.00, *p* = 0.969. BUILD students were equally likely to earn a professional degree as matched non-BUILD students, *b* = 0.02, *SE* = 0.44, *OR* = 1.02, *p* = 0.969. Regarding discipline, only two degrees were earned in non-BBHS disciplines. Both were professional degrees, and one was earned by a BUILD student and the other, a non-BUILD student.

### Research question 3

Are there differences in BUILD doctoral degree outcomes by identities and family educational status compared to those of the matched non-BUILD peers?

**FIGURE 2:**
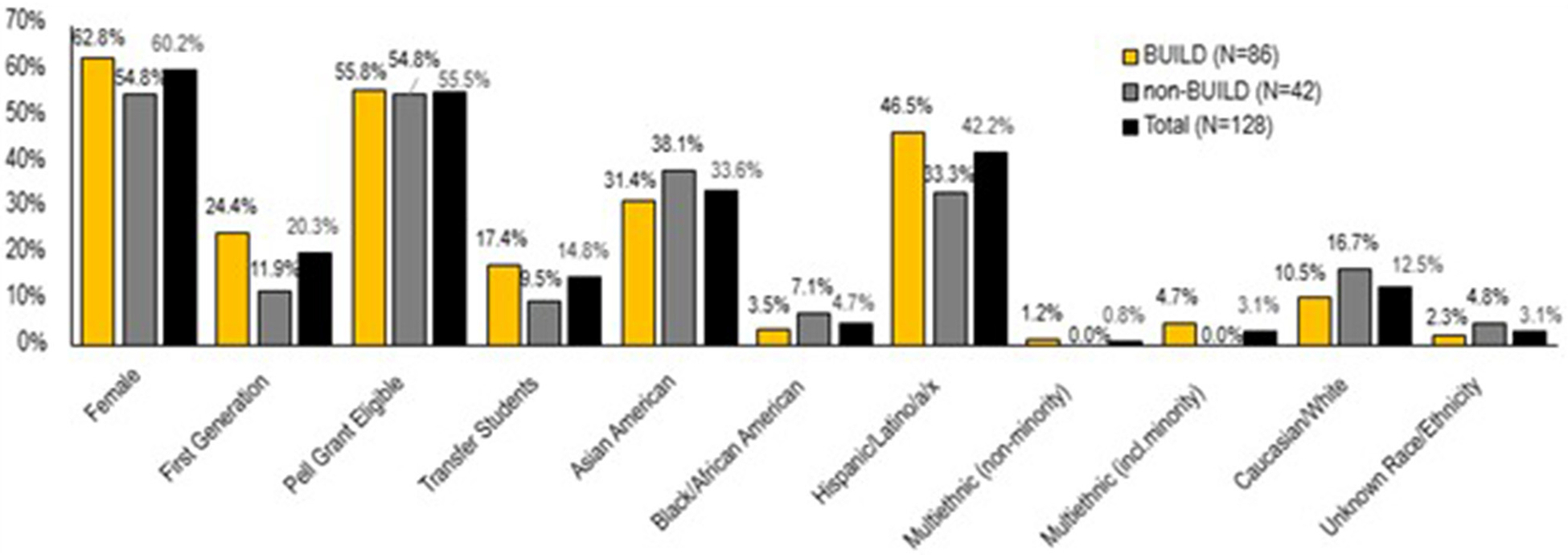
Demographics for students enrolled in doctoral degree programs by BUILD status.

Descriptive statistics summarizing demographics for trainees enrolled in a doctorate program are displayed in Figure 2. Of the 128 doctorate degree program enrollees, a majority were women (60.2%), over half were awarded a Pell-grant as an undergraduate (55.5%), a fifth were first-generation students (20.3%), and 14.8% were undergraduate transfer students. Regarding race/ethnicity, the two largest groups identified as Hispanic/Latinx (42.2%) and Asian American (33.6%). See Figure 2 for demographics for doctoral degree program enrollment disaggregated by BUILD participation status. We caution not to overinterpret differences of percentages, particularly for very small group sizes. Therefore, counts are also reported in parentheses to supplement the percentages. Since some categories had small proportions and/or the group size proportions differ largely between BUILD and non-BUILD groups for some demographics, the power to detect significant differences in enrolling in a doctoral degree program between demographic groups by BUILD status was quite limited. Therefore, inferential tests of interactions were not used to determine if demographic differences of educational outcomes between BUILD and non-BUILD were statistically significant.

When analyzing data by demographic subsamples, logistic regression results in Table 6 revealed that female BUILD students (*p* < 0.001), first-generation BUILD students (*p* = 0.001), Pell-eligible BUILD students (*p* = 0.010), transfer BUILD students (*p* = 0.023) and URM BUILD students (*p* = 0.001) all enrolled in more doctoral programs than their respective non-BUILD counterparts. Notably, the largest effect size was for first generation students ($\left(R_{N}^{2}=0.12\right)$). The odds of enrolling in a doctoral program for first generation BUILD students were 4.79 times the odds for first generation non-BUILD students. Demographic information for Ph.D. and professional degree program enrollments is provided in supplemental materials (Tables 7, 8) as results are comparable to the overall results.

**FIGURE 3:**
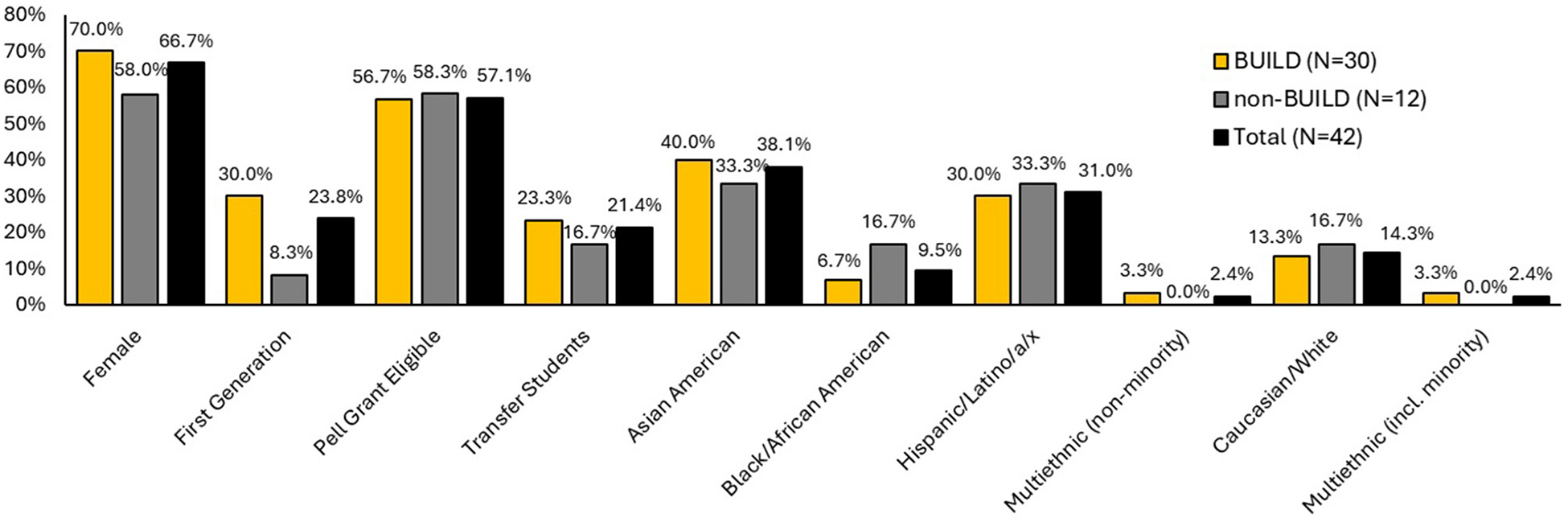
Student demographics for doctorate degree earners by BUILD status.

Descriptive statistics summarizing demographics for doctorate earners are displayed in Figure 3. Of the 42 doctorate degree earners, a majority were women (66.7%), over half were awarded a Pell-grant as an undergraduate (57.1%), about a quarter were first-generation students (23.8%), and about a fifth were undergraduate transfer students (21.4%). Regarding race/ethnicity, again the two largest groups identified as Asian American (40.5%) and Hispanic/Latinx (31.0%). See Figure 3 demographics for doctorate degree earners disaggregated by BUILD participation status. Since the overall group size of doctorate degree earners was small and reasons indicated above, inferential tests of interactions were not used to determine if demographic differences of educational outcomes between BUILD and non-BUILD were statistically significant. When examining subsamples (see Table 9), female BUILD students (*N* = 21, 13%) earned more doctoral degrees than female non-BUILD students (*N* = 7, 4.3%), *X*^2^(1) = 5.76, *p* = 0.016, $R_{N}^{2}=0.04$. The odds of earning a doctoral degree for female BUILD students were 2.79 times the odds for female non-BUILD students, *b* = 1.03, *SE* = 0.45, *OR* = 2.79, *p* = 0.024. Additionally, first generation BUILD students earned more doctoral degrees (*N* = 9, 12.7%) than first generation non-BUILD students (*N* = 1, 1.6%), *X*^2^(1) = 6.76, *p* = 0.009, $R_{N}^{2}=0.12$. The odds of earning a doctoral degree for first generation BUILD students were 8.86 times the odds for first generation non-BUILD students, *b* = 2.18, *SE* = 1.07, *OR* = 8.86, *p* = 0.041. There was not a statistically significant difference between BUILD and non-BUILD students in their doctoral degree attainment for any other demographic category (see Table 9 for full results).

## Discussion

This study aimed to assess the effectiveness of the CSULB BUILD Student Training Programs in providing a direct pipeline to Ph.D. programs. Although other studies have investigated the CSULB BUILD Scholars Program’s outcomes using a propensity score matching approach (Ramos and Vu, 2024; Stormes et al., 2022), this study is the first to examine the longer-term program outcomes of Ph.D. program enrollment and degree attainment with NSC data. Utilizing a quasi-experimental design, the results indicated that BUILD trainees enrolled in doctoral programs at significantly higher rates than their matched non-BUILD peers (26.9% vs. 7.9%). Furthermore, most of the BUILD trainees (93%) pursued doctoral degrees in BBHS-related fields, underscoring the program’s success in aligning with its intended goals. Notably, the odds of earning a Ph.D. were 8.10 times for BUILD students compared to matched non-BUILD students, although there was no significant difference in the attainment of professional degrees. This result likely reflects BUILD/NIH’s programmatic goals including BUILD Programs’ recruitment/selection process that emphasizes Ph.D. degree attainment and actively screens out those who may be solely interested in pursuing professional degrees (e.g., medicine). Still, BUILD students may not disclose their interests in professional degree programs or may not be fully committed to their stated graduate degree goals, especially early on. Lastly, regarding race/ethnicity, enrollment to doctoral programs was greater for BUILD URM students compared to non-BUILD URM students. Although small sample sizes limited the ability to perform interactions and statistical comparisons between all demographic groups, disaggregated data suggest that BUILD students from underrepresented groups enrolled in doctoral programs at higher rates than non-BUILD students including women, first generation, Pell eligible, and transfer students. Regarding doctoral attainment, BUILD had more women and first-generation doctorate earners compared to their non-BUILD counterparts. Furthermore, the largest proportion of degree earners were Asian American and Latine for both BUILD and non-BUILD groups.

Findings from the present study align with what past research has shown on undergraduate research training programs’ effectiveness with supporting the progression of underrepresented groups into STEM research careers (Bayliss et al., 2018; Hall et al., 2016; Vu et al., 2023). Moreover, they provide direct and highly encouraging evidence for the longer-term outcomes that most past research could only suggest or imply. Specifically, the higher doctoral program enrollment and degree attainment rates in BBHS-related disciplines among BUILD students found in this study demonstrate the efficacy of these interventions as a viable pathway for students entering research careers, including for those from historically underrepresented and less resourced groups.

Several limitations should be considered when interpreting these findings. The small sample size of total doctorate earners may limit the generalizability of the results. This study used a variety of covariates (demographic and academic, including research experience) to create the comparison group. However, the quasi-experimental design, while robust, cannot fully eliminate selection bias like randomization. The use of propensity score matching, though effective in creating comparable groups, may not account for all unobserved variables influencing doctoral degree attainment. Moreover, due to the fact that multiple cohorts are included, the timeframe of the data collection gives more time for doctoral program enrollment and degree attainment for students from earlier cohorts. It is also important to note that enrolled students in doctoral programs are continuing their progress toward doctoral attainment. This likely underestimation of the longer-term outcomes suggests that our current results may be a conservative test of our hypotheses. Currently, more BUILD students are enrolled in Ph.D. programs than non-BUILD students (and vice versa for professional degree program enrollments). Therefore, if students persist to completion, there is potential for more of a pronounced difference in type of degree attainment between BUILD and non-BUILD groups as well as other group differences (e.g., race/ethnicity). Both BUILD and non-BUILD students may also continue to enroll in doctoral programs and may also graduate in the future. This may be especially true for disciplines where prospective students are commonly expected to obtain a master’s degree prior to admission to Ph.D. programs. Furthermore, demographic representation of doctoral degree by gender and race/ethnicity largely depends on the discipline of degree. As additional degrees are earned and the sample size grows, future research could turn to examining demographic representation by types of disciplines of degrees. Other outcomes of interest beyond doctoral outcomes could also be examined including outcomes related to enrollment (e.g., applications to graduate school), graduate outcomes like fellowships/awards and publications, and longer-term outcomes like career attainment, though data on these outcomes may be more difficult and costly to collect longer-term.

The present study’s findings can guide institutional priorities and funder efforts aimed at implementing effective practices. That is, our analyses show the value of tracking students in the long term, which requires resources. Having funding for such efforts would allow future research to explore long-term career outcomes of student research training programs, such as the BUILD Program, beyond doctoral degree attainment. These data will allow researchers to draw stronger conclusions about whether such programs do indeed lead to a more diverse BBHS research workforce and which specific program components, such as mentoring, learning communities, and summer research experiences, shape the long-term outcomes.

In conclusion, the CSULB BUILD Student Training Programs have demonstrated significant potential in strengthening the pipeline for Ph.D. attainment as well as fostering diversity within the BBHS research workforce by increasing the representation of underrepresented groups in Ph.D. programs and among Ph.D. degree earners. This study highlights the importance of sustained support for such initiatives to ensure the continued advancement of diversity, equity, and inclusion in the biomedical and behavioral health sciences.

## Figures and Tables

**TABLE 1:** Demographics of BUILD (2015–2018 cohorts) and non-BUILD students before matching.

Demographic (unmatched sample)	BUILD (*N* = 281)		Non-BUILD (*N* = 6310)	
	*N*	Percent	*N*	Percent
First time freshman	221	78.6%	5,771	91.5%
Transfer student	60	21.4%	539	8.5%
First generation student	76	27.0%	1,922	30.5%
Full-time status	277	98.6%	6,087	96.5%
Women	172	61.2%	4,105	65.1%
Men	109	38.8%	2,205	34.9%
African American/Black	14	5.0%	235	3.7%
Asian American	82	29.2%	1,776	28.1%
Caucasian/White	39	13.9%	934	14.8%
Latino/a/x/Hispanic	120	42.7%	2,620	41.5%
Native American	0	0.0%	12	0.2%
Pacific Islander	0	0.0%	14	0.2%
Multiethnic including minority	7	2.5%	198	3.1%
Multiethnic not including minority	9	3.2%	106	1.7%
College of Business	0	0.0%	301	4.8%
College of Education	0	0.0%	80	1.3%
College of Engineering	65	23.1%	908	14.4%
College of Health and Human Sciences	45	16.0%	1,184	18.8%
College of Liberal Arts	71	25.3%	965	15.3%
College of Natural Sciences & Mathematics	97	34.5%	720	11.4%
College of the Arts	0	0.0%	369	5.8%
Undeclared	3	1.1%	1,783	28.20%
Pell eligible	165	58.7%	3558	56.4%
Age at CSULB entry	*M* = 19.13 (SD = 3.49)	16–45 years	*M* = 18.33 (SD = 2.40)	16–62 years

Minimum and maximum reported for age at CSULB entry rather than percentage.

**TABLE 2:** Relationships between potential covariates, BUILD participation, and academic outcomes pre-matching and post-matching.

Covariate	Pre-Matching			Post-Matching
	BUILD (1/0)	Graduated (1/0)	Last GPA	BUILD (1/0)
	Cohen’s *d*, p-value	Cohen’s *d, p*-value	*r/d, p*-value	Cohen’s *d, p*-value
Gender (1 = Woman)	−*0.08, p = 0.197*	*0.04, p = 0.135*	***0.31, p*** < ***0.001***	−0.02, *p* = 0.884
African American/Black	*0.07, p = 0.342*	−0.01, *p* = 0.756	−**0.34, *p*** < **0.001**	0.03, *p* = 0.761
Asian American	0.02, *p* = 0.706	*0.03, p* = *0.178*	***0.27, p*** < ***0.001***	−0.07, *p* = 0.407
Caucasian/White	−0.03, *p* = 0.670	*0.07, p* = *0.006*	***0.30, p* < *0.001***	0.03, *p* = 0.717
Latino/a/x/Hispanic	0.02, *p* = 0.694	−*0.09, p* < *0.001*	−***0.31, p* < *0.001***	0.07, *p* = 0.472
Multiethnic including minority	−0.04, *p* = 0.541	0.01, *p* = 0.683	0.08, *p* = 0.268	−0.01, *p* = 0.914
Multiethnic not including minority	***0.12, p = 0.154***	−*0.04, p = 0.104*	−***0.19, p*** = ***0.102***	0.09, *p* = 0.293
Unknown Race/Ethnicity	0.00, *p* = 0.838	*0.05, p* = *0.041*	**0.13, *p*** = **0.098**	−0.04, *p* = 0.648
Age at CSULB entry	***0.32, p*** < **0.001**	***0.41, p*** < ***0.001***	−0.02, *p* = 0.099	0.01, *p* = 0.880
Pell Eligibility (1 = Yes)	0.05, *p* = 0.440	−*0.08, p = 0.001*	−**0.20, *p*** < **0.001**	0.01, *p* = 0.897
Pell Eligibility (1 = Missing Pell)	***0.16, p*** = ***0.054***	***0.32, p*** < ***0.001***	−0.05, *p* = 0.525	0.02, *p* = 0.803
First Generation (1 = Yes)	−*0.07, p = 0.210*	−0.01, *p* = 0.588	−**0.23, *p*** < **0.001**	−0.01, *p* = 0.947
Year of Matriculation	−**0.62, p** < **0.001**	−***1.59, p*** < ***0.001***	−0.02, *p* = 0.137	−0.07, *p* = 0.454
Full Time Status (1 = Yes)	***0.16, p*** < ***0.001***	*0.04, p = 0.108*	***0.40, p*** < ***0.001***	−0.04, *p* = 0.692
Transfer Student (1 = Yes)	***0.45, p*** < ***0.001***	***0.59, p*** < ***0.001***	0.08, *p* = 0.010	0.05, *p* = 0.585
College of Engineering	***0.25, p*** = ***0.001***	0.00, *p* = 0.863	−***0.12, p*** < ***0.001***	0.04, *p* = 0.679
College of Health and Human Sciences	−*0.07, p = 0.222*	*0.09, p* = *0.001*	***0.43, p*** < ***0.001***	−0.06, *p* = 0.542
College of Liberal Arts	***0.27, p*** < ***0.001***	***0.31, p*** < ***0.001***	0.06, *p* = 0.101	0.02, *p* = 0.788
College of Natural Sciences and Mathematics	***0.71, p*** < ***0.001***	−*0.06, p = 0.011*	−**0.13, *p*** < **0.001**	−0.01, *p* = 0.865
College of Arts	−***0.24, p*** < ***0.001***	−***0.26, p*** < ***0.001***	**0.36, *p*** < **0.001**	0.08, *p* = 0.369
Undecided major	−***0.24, p*** < ***0.001***	−***0.49, p*** < ***0.001***	−***0.68, p*** < ***0.001***	−0.02, *p* = 0.830
GPA at CSULB entry	***0.22, p*** < ***0.001***	0.00, *p* = 0.954	**0.49, *p*** < **0.001**	0.03, *p* = 0.743
Enrolled in independent research (1 = Yes)	***3.08, p*** < ***0.001***	***0.41, p*** < ***0.001***	***0.56, p*** < ***0.001***	*0.09, p* = *0.296*

Bolded if Cohen’s *d* > 0.10 or *r* > 0.10 regardless of statistical significance; italicized if non-parametric test (Welch’s *t*-test) was used, positive values indicate BUILD was higher on average than non-BUILD group on associated covariate for Cohen’s *d*.

**TABLE 3:** Logistic regression predicting BUILD participation.

Covariates for propensity scores	*b*	*SE*	*p*	Odds ratio
Gender (1 = Woman)	−0.34	0.181	0.059	0.71
African American/Black	0.48	0.419	0.252	1.62
Asian American	0.62	0.248	0.012	1.86
Latino/a/x/Hispanic	0.77	0.239	0.001	2.15
Multiethnic including minority	0.16	0.513	0.760	1.17
Multiethnic not including minority	1.69	0.560	0.002	5.43
Unknown Race/Ethnicity	0.25	0.485	0.612	1.28
Age at CSULB entry	0.01	0.030	0.636	1.01
Pell eligibility (1 = Yes)	0.33	0.178	0.064	1.39
Missing pell eligibility (1 = Missing)	0.45	0.428	0.290	1.57
First generation (1 = Yes)	−0.08	0.190	0.688	0.93
Year of CSULB entry	−0.03	0.005	<0.001	0.97
Full time status (1 = Yes)	1.71	0.798	0.032	5.53
Transfer student (1 = Yes)	0.61	0.298	0.039	1.85
College of Engineering	0.33	0.247	0.182	1.39
College of Health and Human Sciences	0.06	0.244	0.813	1.06
College of Natural Sciences and Mathematics	0.09	0.217	0.684	1.09
College of Arts	−1.84	1.050	0.079	0.16
College: Undecided	−0.70	0.769	0.363	0.50
GPA at CSULB entry	0.45	0.244	0.069	1.56
Enrolled in independent research (1 = Yes)	4.15	0.200	<0.001	63.66

Referent group for Race/ethnicity was White/Caucasian, Referent group for college was Liberal Arts, Missing Pell variable was included to retain students that were missing this information, Referent group was not Pell Eligible, Odds Ratio of 1 indicates BUILD participation and non-BUILD participation were equally likely.

**TABLE 4:** Counts and odds of doctoral enrollment by BUILD status and type of degree.

Group	Total enroll	No enroll	Odds enroll	Ph.D. enroll	No Ph.D. enroll	Odds Ph.D. enroll	Prof. degree enroll	No Prof. degree enroll	Odds Prof. degree enroll
Total (*N* = 495)	128	367	0.35	88	407	0.22	40	455	0.09
BUILD (*N* = 268)	86	182	0.47	71	197	0.36	15	253	0.06
Non-BUILD (*N* = 227)	42	185	0.23	17	210	0.08	25	202	0.12
			Odds ratio: 2.08***			Odds ratio: 4.45***			Odds ratio: 0.48*

Enroll, Enrollment; Prof., Professional degrees.

* *p* < 0.05 and

*** *p* < 0.001

**TABLE 5:** Counts and odds of doctoral degrees by BUILD status and type of degree.

Group	Total degrees	No degrees	Odds degree	Ph.D.	No Ph.D.	Odds Ph.D.	Prof. degree	No Prof. degree	Odds Prof. degree
Total (*N* = 495)	42	453	0.09	20	475	0.04	22	473	0.05
BUILD (*N* = 268)	30	238	0.13	18	250	0.07	12	256	0.05
Non-BUILD (*N* = 227)	12	215	0.06	2	225	0.01	10	217	0.05
			Odds ratio: 2.26*			Odds ratio: 8.10***			Odds ratio: 1.02

Prof., Professional degrees;

* *p* < 0.05 and

*** *p* < 0.001.

**TABLE 6:** Logistic regression model results for doctoral program enrollment regressed on BUILD status by subsample.

Subsample	Doctoral Degrees Earned		*X*^2^ Omnibus Test	$R_{N}^{2}$	*b*(SE)	Exp(*B*)	Wald *p*
	BUILD	Non-BUILD					
Women (*N* = 298)	54/161 (33.5%)	23/137 (16.8%)	*X*^2^(1) = 11.13, *p* < 0.001	0.05	0.92(0.28)	2.50**	0.001
First generation (*N* = 133)	21/71 (29.6%)	5/62 (8.1%)	*X*^2^(1) = 10.44, *p* = 0.001	0.12	1.57(0.53)	4.79**	0.003
Pell eligible (*N* = 290)	48/158 (30.4%)	23/132 (17.4%)	*X*^2^(1) = 6.67, *p* = 0.010	0.03	0.73(0.29)	2.07*	0.011
Transfer (*N* = 101)	15/57 (26.3%)	4/44 (9.1%)	*X*^2^(1) = 5.15, *p* = 0.023	0.08	1.27(0.61)	3.57*	0.035
URM (*N* = 243)	47/138 (34.1%)	17/105 (16.2%)	*X*^2^(1) = 10.18, *p* = 0.001	0.06	0.98(0.32)	2.67**	0.002

* *p* < 0.05 and

** *p* < 0.01.

**TABLE 7:** Student demographics for enrolled in professional program by BUILD status.

Demographic	BUILD (*N* = 14)	Non-BUILD (*N* = 24)	Overall (*N* = 38)
	% (Total)	% (Total)	% (Total)
First generation	35.7% (5)	0.0% (0)	13.2% (5)
Pell grant eligible	50.0% (7)	58.3% (14)	55.3% (21)
Transfer students	21.4% (3)	12.5% (3)	15.8% (6)
Gender			
Female	71.4% (10)	54.2% (13)	60.5% (23)
Male	28.6% (4)	45.8% (11)	39.5% (15)
Race/ethnicity			
Asian American	64.3% (9)	41.7% (10)	50.0% (19)
Black/African American	0.0% (0)	8.3% (2)	5.3% (2)
Hispanic/Latino/a/x	28.6% (4)	29.2% (7)	28.9% (11)
Caucasian/White	7.1% (1)	20.8% (5)	15.8% (6)

**TABLE 8:** Student demographics for enrolled in PhD program by BUILD status.

Demographic	BUILD (*N* = 72)	Non-BUILD (*N* = 18)	Overall (*N* = 90)
	% (Total)	% (Total)	% (Total)
First generation	22.2% (16)	27.8% (5)	23.3% (21)
Pell grant eligible	56.9% (41)	50.0% (9)	55.6% (50)
Transfer students	16.7% (12)	5.6% (1)	14.4% (13)
Gender			
Female	61.1% (44)	55.6% (10)	60.0% (54)
Male	38.9% (28)	44.4% (8)	40.0% (36)
Race/ethnicity			
Asian American	25.0% (18)	33.3% (6)	26.7% (24)
Black/African American	4.2% (3)	5.6% (1)	4.4% (4)
Hispanic/Latino/a/x	50.0% (36)	38.9% (7)	47.8% (43)
Multiethnic (non-minority)	1.4% (1)	0% (0)	1.1% (1)
Multiethnic (incl.minority)	5.6% (4)	0% (0)	4.4% (4)
Caucasian/White	11.1% (8)	11.1% (2)	11.1% (10)
Unknown Race/Ethnicity	2.8% (2)	11.1% (2)	4.4% (4)

**TABLE 9:** Logistic regression model results for doctoral degree attainment regressed on BUILD status by subsample.

Subsample	Doctoral Degrees Earned		*X*^2^ Omnibus Test	$R_{N}^{2}$	*b*(SE)	Exp(*B*)	Wald *p*
	BUILD	Non-BUILD					
Women (*N* = 298)	21/161 (13%)	7/137 (4.3%)	*X*^2^(1) = 5.76, *p* = 0.016	0.04	1.03 (0.45)	2.79*	0.024
First generation (*N* = 133)	9/71 (12.7%)	1/62 (1.6%)	*X*^2^(1) = 6.76, *p* = 0.009	0.12	2.18 (1.07)	8.86*	0.041
Pell eligible (*N* = 290)	17/158 (10.8%)	7/132 (5.3%)	*X*^2^(1) = 2.93, *p* = 0.087		0.77 (0.47)	2.15	0.100
Transfer (*N* = 101)	7/57 (12.3%)	2/44 (4.5%)	*X*^2^(1) = 1.96, *p* = 0.161		1.08 (0.83)	2.94	0.193
URM (*N* = 243)	12/138 (8.7%)	6/105 (5.7%)	*X*^2^(1) = 0.79, *p* = 0.374		0.45 (0.32)	1.57	0.383

* *p* < 0.05.

## Data Availability

The data analyzed in this study is subject to the following licenses/restrictions: The dataset includes sensitive information including school records over multiple institutions that could identify the subjects of this research study, especially for individuals from underrepresented minority groups given the specificity of the information including demographics and school record information even when the data are scrubbed of names and ID numbers. Requests to access these datasets should be directed to Erin H. Arruda at Erin.Arruda@csulb.edu.

## References

[R1] Abeywardana SU, Velasco S, Hall N, Dillon J, and Chun C-A (2020). Nearpeer mentoring in an undergraduate research training program at a large master’s comprehensive institution. Understand. Interven 11:12477. PMC9094756 35558987

[R2] Bai H (2011). Using propensity score analysis for making causal claims in research articles. Educ. Psychol. Rev 23, 273–278. doi: 10.1007/s10648-011-9164-9

[R3] Bai H, and Clark MH (2019). Propensity Score Methods and Applications. Thousand Oaks, CA: Sage. doi: 10.4135/9781071814253

[R4] Bayliss F, Peterfreund A, and Rath K (2018). “Programmatic mentoring,” in Mentoring at Minority Serving Institutions (MSIs): Theory, Design, Practice and Impact (eds) McClinton J, Mitchell DS, Hughes GB, and Melton MA (Charlotte, NC: Information Age Publishing, Inc.).

[R5] Bennett CL, Salinas RY, Locascio JJ, and Boyer EW (2020b). Two decades of little change: an analysis of US medical school basic science faculty by sex, race/ethnicity, and academic rank. PLoS ONE 15:e0235190. doi: 10.1371/journal.pone.0235190 32735593 PMC7394429

[R6] Bennett J, Lattuca L, Redd K, and York T (2020a). Strengthening Pathways to Faculty Careers in STEM: Recommendations for Systemic Change to Support Underrepresented Groups. Lessons from the APLU INCLUDES Project. Association of Public and Land-grant Universities. Available at: https://www.aplu.org/wp-content/uploads/strengthening-pathways-to-faculty-careers-in-stem-recommendations-for-systemic-change-to-support-underrepresented-groups.pdf.

[R7] Cochran WG, and Rubin DB (1973). Controlling bias in observational studies: a review. Sankhyā Ind. J. Statistics Series A 35, 417–446.

[R8] Hall AK, Miklos A, Oh A, and Gaillard SD (2016). Educational Outcomes from the Maximizing Access to Research Careers Undergraduate Student Training in Academic Research (MARC U-STAR) Program. Available at: https://www.nigms.nih.gov/about/dima/Documents/MARC-paper031416.pdf (accessed 10 July 2022).

[R9] IBM Corp (2023). IBM SPSS Statistics for Windows, Version 29.0.2.0 Armonk, NY: IBM Corp

[R10] Kingsford L, Mendoza R, Dillon J, Chun C-A, and Vu K-P (2022). Broadening and diversifying the behavioral and biomedical research workforce through early access to an undergraduate research training program. Understand. Interven 13, 1–24. PMC10358370 37475728

[R11] Linn MC, Palmer E, Baranger A, Gerard E, and Stone E (2015). Undergraduate research experiences: impacts and opportunities. Science 347, 627–633. doi: 10.1126/science.1261757 25657254

[R12] Ramos HV, and Vu K-PL (2024). Research, science identity, and intent to pursue a science career: a BUILD intervention evaluation at CSULB. Educ. Sci 14:647. doi: 10.3390/educsci14060647 PMC11356325 39206225

[R13] Ransdell LB, Lane TS, Schwartz AL, Wayment HA, and Baldwin JA (2021). Mentoring new and early-stage investigators and underrepresented minority faculty for research success in health-related fields: An integrative literature review (2010–2020). Int. J. Environ. Res. Public Health 18:432. doi: 10.3390/ijerph18020432 33430479 PMC7826619

[R14] Rosenbaum PR, and Rubin DB (1985). Constructing a control group using multivariate matched sampling methods that incorporate the propensity score. Am. Stat 39, 33–38. doi: 10.1080/00031305.1985.10479383

[R15] Stormes K, Streicker NA, Bowers GK, Ayala P, and Urizar G (2022). Impact of undergraduate research training programs: an illustrative example of finding a comparison group and evaluating academic and graduate school outcomes. Assessment 5, 25–36. doi: 10.18833/spur/5/3/8 PMC10002406 36909939

[R16] Vu K-P, Mendoza R, Chun C-A, Dillon J, and Kingsford L (2023). The CSULB BUILD Scholars program: a research intensive upper-division program to broaden and diversify the behavioral and biomedical research workforce. Understand. Interven 14, 1–37. PMC11368045 39224136

[R17] Whittinghill JC, Slovacek SP, Flenoury LP, and Miu V (2019). A 10-year study on the efficacy of biomedical research support programs at a public university. Scholarship Pract. Undergrad. Res 3, 30–38. doi: 10.18833/spur/3/1/3

[R18] Wyss CJ, LoCasale RJ, Brookhart AM, and Stürmer T (2013). Variable selection for propensity score models when estimating treatment effects on multiple outcomes: a simulation study. Pharmacoepidemiol. Drug Saf 22, 77–85. doi: 10.1002/pds.3356 23070806 PMC3540180

